# Lambda CI Binding to Related Phage Operator Sequences Validates Alignment Algorithm and Highlights the Importance of Overlooked Bonds

**DOI:** 10.3390/genes14122221

**Published:** 2023-12-15

**Authors:** Jacklin Sedhom, Lee A. Solomon

**Affiliations:** Department of Chemistry and Biochemistry, George Mason University, Fairfax, VA 22030, USA; jyassa@gmu.edu

**Keywords:** DNA-protein binding, bacteriophage, genetic regulation, λ-phage

## Abstract

Bacteriophage λ’s CI repressor protein controls a genetic switch between the virus’s lysogenic and lytic lifecycles, in part, by selectively binding to six different DNA sequences within the phage genome—collectively referred to as operator sites. However, the minimal level of information needed for CI to recognize and specifically bind these six unique-but-related sequences is unclear. In a previous study, we introduced an algorithm that extracts the minimal direct readout information needed for λ-CI to recognize and bind its six binding sites. We further revealed direct readout information shared among three evolutionarily related lambdoid phages: λ-phage, Enterobacteria phage VT2-Sakai, and Stx2 converting phage I, suggesting that the λ-CI protein could bind to the operator sites of these other phages. In this study, we show that λ-CI can indeed bind the other two phages’ cognate binding sites as predicted using our algorithm, validating the hypotheses from that paper. We go on to demonstrate the importance of specific hydrogen bond donors and acceptors that are maintained despite changes to the nucleobase itself, and another that has an important role in recognition and binding. This in vitro validation of our algorithm supports its use as a tool to predict alternative binding sites for DNA-binding proteins.

## 1. Introduction

**Figure 1:**
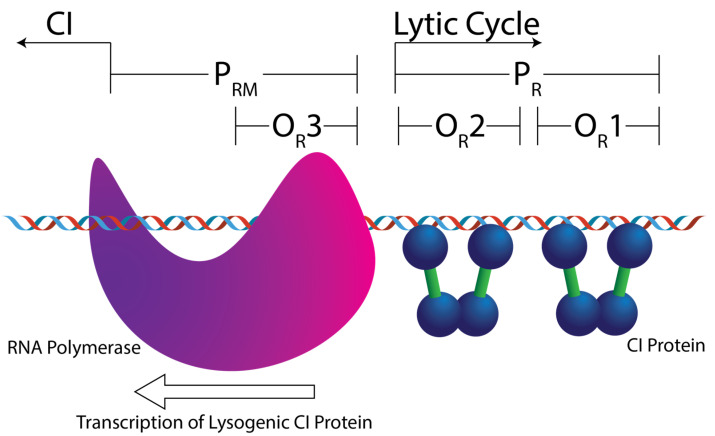
Cartoon depicting the right side of a simplified bacteriophage operator region, the O_L_ region has been removed for clarity. The repressor protein (CI) binds to three sites in the phage genome: O_R_1, O_R_2, and O_R_3. When CI is bound to O_R_1 and O_R_2, it activates transcription of the *c*I gene from P_RM_, shown here by the presence of RNA polymerase. These two sites also form the P_R_ promotor of lytic cycle genes. Removal of CI opens this promotor up to RNA polymerase. If O_R_1–3 are occupied then no viral genes are transcribed.

Lambdoid phages are a group of temperate viruses that infect *Escherichia coli*, some of which cause significant health issues through Shiga Toxin (Stx) expression, which can lead to hemolytic uremic syndrome [1,2]. Upon infection of the bacterium, the virus can either integrate its genome into the host’s genome and remain silent, in what is named the lysogenic lifecycle, or they can replicate and lyse the bacterial cell, in what is named the lytic lifecycle [3,4,5,6,7,8]. The lytic–lysogenic decision-making process is partly controlled by a phage-encoded transcriptional repressor protein, CI [9]. CI’s regulatory activity stems, in part, from its ability to recognize six different DNA sequences in the bacteriophage genome, which are embedded in genetic promoters collectively named the “operator” site [7]. CI binding to the P_R_ promoter suppresses the production of lytic lifecycle proteins (Figure 1) [10]. However, when CI is bound to P_R,_ it activates transcription of the *c*I gene from a similarly embedded promoter named P_RM_. Thus, the CI protein functions as both a transcriptional repressor and activator. When the concentration of CI protein is high enough, it binds to O_R_3 and represses transcription from P_RM_. At this stage, all phage activity will be halted.

**Figure 2:**
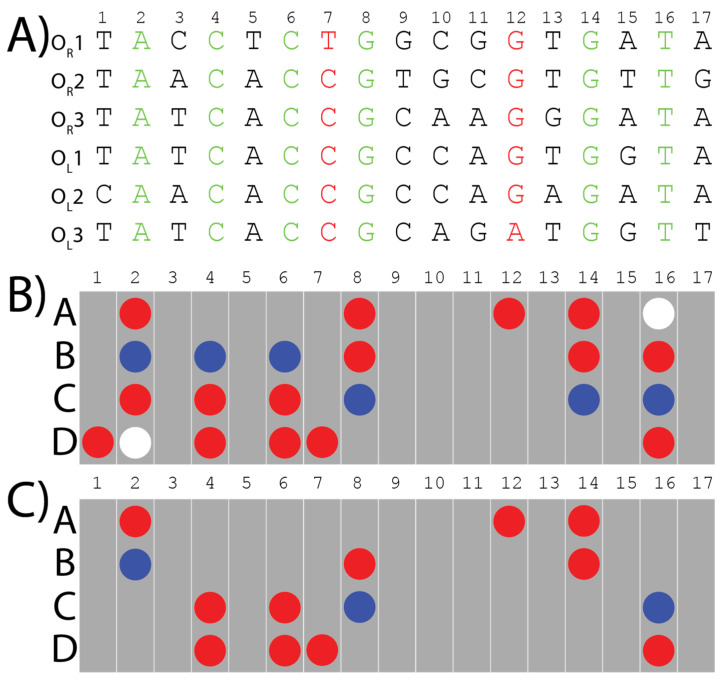
The six binding sites of λ-CI to which the algorithm was applied, to extract the consensus pattern and the refined distinct pattern: (**A**) The six binding sites’ sequences are color coded. Black letters are the base pairs that are different among the six binding sites and don’t have any bonds in common. Green letters are the conserved base pairs which have direct bonds and contacts with the CI-protein shared among the six binding sites. Red letters are different base pairs in the six binding sites which contribute with the same bonds to bind with λ-CI repressor. (**B**) The consensus pattern of the bonds and interactions extracted from the six λ-phage operator sequences using our algorithm. Red circles are hydrogen bond acceptors, blue circles are hydrogen bond donors, and white circles are methyl groups. Each grey bar represents one base pair. Rows A and B represent the major groove interactions from bases on the 5′-3′ strand whereas rows C and D represent the major groove interactions from bases on the compliment 3′-5′ strand. (**C**) The final distinct pattern of the bonds and the interactions shared among the six λ-phage operator sites, verified using the corresponding crystal structures.

The six operator sites have different sequences (Figure 2A), but CI can still specifically identify them within the greater *E. coli* genome with high selectivity. Despite years of investigations into λ-phage genetics, a significant question remains: what is the minimal level of information required for multi-site selectivity, and which specific interactions promote binding versus specificity of these six sites over others? This is what we are investigating.

To answer this question, we previously developed an algorithm that can extract the minimum direct readout information required for a protein to recognize multiple binding sites [4]. In short, our algorithm converts the information presented in the major groove of the input sequences into a two-dimensional array. The length is based on that of the input sequence and the height is composed of four rows (A, B, C, and D) indicating a hydrogen bond donor, acceptor, or methyl group exposed in the major groove representative of the nucleobase (Figure 2B,C). In this organizational scheme, the 5′-to-3′ strand provides positions A and B, whereas the compliment 3′-to-5′ strand provides positions C and D. The algorithm then aligns these arrays of non-covalent interactions to identify what information is being maintained amongst multiple DNA sequences, which is called “the consensus pattern” (Figure 2B). To ensure the contacts we extract from these patterns are relevant to protein binding, we consult published structures for further refinement of the consensus pattern. If a contact is maintained in the DNA consensus pattern but is not contacting the protein in the published structure (e.g., due to it being on the opposite side of the DNA due to rotation) it is removed from the consensus pattern to produce the final pattern, which is called “the distinct pattern” (Figure 2C). We hypothesize the distinct pattern is the minimal required information for site-specific recognition. Importantly, the algorithm can reveal if key interacting hydrogen bonds are maintained even if the nucleobase it comes from is different. Interactions that may have been overlooked when using alignment methods focused on nucleobase identity [11,12,13]. Analyses of the six λ-phage operator sites made using Clustal Omega only identified the six conserved bases (Green nucleotides in Figure 2A) [14,15], whereas an EMBOSS Cons analysis returned the following sequence: TAtCACCGCcaGTGaTA, where the lowercase letters indicate there was no consensus [14,16]. While both analyses are useful, this does not help to identify how the proteins interact with the DNA. Recent work by Lin and Guo has shown that which strand the protein contacts is important and should be a part of this analysis [17]. Thus, our algorithm extracts the specific interactions made between the protein and DNA, which can help identify if one strand is making more or less contact with the protein and potentially identify additional contacts.

We applied our algorithm to the six binding sites of λ-CI, to form a consensus pattern (Figure 2B). This pattern was refined from the crystal structure of a λ-CI binding complex to extract what we call the “distinct” pattern, which represents the minimal information required by λ-CI to recognize its binding sites [4]. The distinct pattern of λ-CI comprises 14 hydrogen bonds from conserved base pairs in the six binding sites except for two Purine N7-lone pair hydrogen bonds at locations 7D and 12A, which are maintained despite coming from a different nucleobase (Figure 2C). We hypothesized that these two bonds might have a role in recognition from λ-CI.

In that same study, this analysis was applied to the binding sites of the three evolutionarily related members of the lambdoid phages—λ-phage, Enterobacteria phage VT2-Sakai (VT2-SA), and Stx2 converting phage I (Stx2I)—to reveal the hidden information that might be shared among them during evolution [5]. These three phages all contain six operator sites that function similarly to λ-phage. The preliminary results show considerable information shared among the three phages, specifically at the binding site O_R_3. As a result, we hypothesized that the CI repressor of λ-phage could recognize and bind the operator sites of either VT2-SA or Stx2I [4]. 

In this present study, we sought to validate the algorithm’s hypotheses and efficacy. We use electrophoretic mobility shift assays (EMSAs) to show the binding between λ-CI and the individual DNA operator sequences of the phages: λ-phage, VT2-SA, and Stx2I as a function of their overlap predicted using our algorithm [18]. In addition, we sought to investigate if these two bonds 7D and 12A are important for λ-CI binding as hypothesized. Our analysis revealed the importance of 12A hydrogen bond acceptor over 7D hydrogen bond acceptor in recognition and binding. We also sought to extend our analysis to include all 14 bonds of the distinct pattern to see which specific bonds are important for protein–DNA binding. Thus, we looked for these 14 distinct bonds in the 12 operator sites of phages VT2-SA and Stx2I. Our results revealed that the hydrogen bond acceptor at position 6D is important for λ-CI binding, however 7D may play a role in compensating for alterations at this position. 

In vitro experiments confirm that λ-CI can bind the other two phages’ operator sites as predicted. In silico analysis reveals that the binding sites recognized by λ-CI with relatively high affinity maintained at least one of the hydrogen bonds in locations 6D and 12A and at least 50% overlap with the distinct patterns of λ-CI. Our findings also emphasize the significance of these key interactions for λ-CI binding and support the results of our algorithm, and its ability to predict whether or not a DNA-binding protein recognizes a certain DNA sequence. 

## 2. Materials and Methods

### 2.1. Chemicals

All chemicals were purchased from Millipore Sigma and Thermofisher Scientific unless otherwise noted. Polyacrylamide and related gel supplies were purchased from BioRad. 

### 2.2. Sequence Analysis

Clustal Omega and EMBOSS Cons analyses were both performed by accessing the tools from the EMBL Bioinformatics Institute’s webpage [14]. In both cases, the default parameters were used.

### 2.3. Protein Purification

The method was adapted from Solomon et al. [19] and Gao et al. [20]. The protein used in this paper was obtained as follows. The gene for λ-CI was ordered from Twist Biosciences. Embedded in the pET 29b+ plasmid construct containing a His-tag, a TEV cleavage site containing a linker, and the protein of interest. Upon delivery, BL-21 DE3 strain *E. coli* cells were transformed with this plasmid DNA. The BL-21 cells were grown in TB media at 37 °C until the OD600 was at 0.6 AU. Overexpression of our gene was induced via the addition of 0.5 mM IPTG. The cells were further incubated for 5 h at 37 °C and then were harvested via centrifugation using a Beckman Coulter Avanti J-E centrifuge with a JLA-10.500 rotor. The pellet was then resuspended in resuspension buffer (500 mM NaCl, 20 mM NaH_2_PO_4_, 20 mM Imidazole, pH 7.4) and sonicated to lyse the cells. The lysate was spun down for 1 h at 20,000 relative centrifugal force (rcf) using a JA-20 rotor. The supernatant was then gravity-fed through a Ni-NTA column. Then, the column was washed with five column volumes of the resuspension buffer and a step gradient from resuspension buffer to elution buffer (500 mM NaCl, 20 mM NaH_2_PO_4_, 500 mM Imidazole, pH 7.4) was used to elute the protein. Each step was one column volume and went from 0% elution buffer (500 mM NaCl, 20 mM NaH_2_PO_4_, 500 mM Imidazole, pH 7.4) to 100% in steps of 10%. The flow-through was dialyzed into TEG buffer (50 mM Tris-base, 0.1 mM EDTA, 150 mM NaCl and 10% glycerol, pH 7.5). Its concentration was determined using the absorbance at 280 nm with an extinction coefficient of 22,500 M^–1^ cm^–1^. The extinction coefficient was determined with the ProtParam tool on the ExPASy website (http://web.expasy.org/protparam/, accessed on 1 February 2022). The protein solution was then fractioned into small aliquots and stored at −20 °C. 

### 2.4. Electrophoretic Mobility Shift Assays (EMSAs)

All the EMSAs were performed using 10% polyacrylamide gels at 150 V at room temperature. The electrophoresis buffer used was 1× TBE (0.089 M Tris base, 0.089 M Boric acid, 0.002 M EDTA, free acid, pH 8.3) (VWR Life Science). A serial dilution was prepared for λ-CI protein with a final concentration of 0.4, 0.8, 2, 4, 8, 16, 32, and 40 μM using HEPES buffer (10 mM HEPES, 150 mM NaCl, pH 7.4) to bind 50 nM of each of the DNA sequences. The DNA solution used in binding was diluted using HEPES buffer as well. The DNA oligonucleotides of all 18 sequences (the six operator sequences for each of the following phages: λ, VT2-SA, and Stx2I) were purchased from Integrated DNA Technologies company. Overhangs where also added on each side to improve DNA stability. The overhang sequence on the 5′ end of the oligo is 5′-GGTTATTATGG-3′, and the overhang sequence on the 3′ side is 5′-TGCAAGTGC-3′. These were present and consistent on all 18 oligos (Table S1). The protein–DNA complexes were incubated for 20 min at room temperature before the addition of 1× gel loading dye with no SDS (Bio-Rad, Hercules, CA, USA). They were loaded into the polyacrylamide gel to run. To visualize the DNA on the gels, all the gels were stained using SYBR Safe DNA gel stain (Invitrogen, Waltham, MA, USA). 

### 2.5. Data Analysis

All the DNA-stained bands on the gels were detected using G: BOX BioImaging Systems (Syngene). The data analysis method was adapted from Heffler et al. [21]. ImageJ software (version 1.46r), which is freely downloadable from NIH [22], was used to quantify the signal in each DNA band to calculate the apparent equilibrium dissociation constant (*K_Dapp_*) values of the binding. The fraction of DNA that was bound to the protein was determined using the following equation:

(1) $$\text{Fraction Bound}=\frac{\text{Bound DNA}}{\text{Bound DNA}+\text{Free DNA}}$$

Then, the fraction bound of DNA was plotted versus the different concentrations λ-CI that revealed signals in the EMSA gels only. The equation

(2) $$\text{Fraction Bound}=B_{max}\left(\frac{\left[\lambda \cdot CI\right]}{K_{Dapp}+[\lambda \cdot CI]}\right)$$

was used to fit the data, where *B_max_* represents the maximum binding and [*λ-CI*] represents the protein concentration [21]. The *K_Dapp_* values were obtained using curve_fit from the SciPy package in Python after fitting the curve [23].

It is important to mention, that we are only reporting apparent dissociation constant (*K_Dapp_*) values in this paper due to the low resolution of sub-micromolar concentration DNA bands in EMSA experiments. Ream et al. have reported that EMSA’s are only capable of providing non-equilibrium binding values (i.e., an apparent *K_D_*) [24]. 

### 2.6. Pattern Alignment

The genomic sequences of λ-phage, VT2-SA phage, and Stx2I phage’s binding sites are available online in the NCBI taxonomy database [25,26]. The table with all of the DNA sequences used in this paper can be found in Supplementary Table S1. Our algorithm was used, in a previous study, to align the binding sites of λ-phage and to create the consensus pattern as described in [4]. The same algorithm was used to align the λ-phage’s consensus pattern with each of the 12 binding sites of VT2-SA and Stx2I phages to extract the matching pattern for each binding site. The algorithm calculated the percent match for each of the 12 binding sites with the consensus pattern of λ-phage. In addition, 12 aligned patterns were created from this step (all the codes are provided in the GitHub repository).

Previously, we reported the distinct binding pattern of λ-phage after refining its consensus pattern from the crystal structures. This λ-phage’s distinct pattern was used to refine the 12 aligned patterns, so we can calculate the percentage of matching for each of the 12 binding sites with the distinct pattern of λ-phage’s binding sites.

The 12 refined patterns were expressed in a map showing the absence and the presence of each of the 14 distinct bonds in each of these 12 binding sites after ranking them based on their *K_Dapp_* values from the lowest to the highest. 

## 3. Results

Our previous study predicted binding between λ-CI and the six binding sites of these other two lambdoid bacteriophages: VT2-SA and Stx2I. These two other bacteriophages were chosen based on their evolutionary relationship to λ, taken from the work of Glazko et al. who show a phylogenetic tree of bacteriophages. We only considered other phages in the same clade as λ. Of these seven phages, we selected two that had operator regions with a similar architecture (i.e., six distinct operator sites split into three O_R_ and three O_L_ sites) For example, 933W was excluded because it only has five operator sites. We also only selected phages whose operator sequences were 17 base pairs in length, similar to λ-phage [27]. 

**Figure 3:**
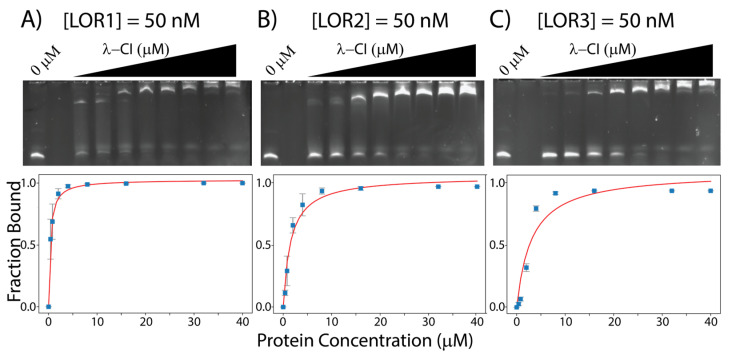
EMSAs gels for λ-CI with (**A**) 50 nM LOR1, (**B**) 50 nM LOR2, and (**C**) 50 nM LOR3. All the binding data were measured using ImageJ and curve fitted using python. Gels for LOL1, LOL2, and LOL3 are shown in Figure S1 in Supplementary Data.

To test our hypothesis that the λ-CI protein could bind to all 18 DNA-sequences (i.e., the six operator sequences from λ, VT2-SA and Stx2-I), we ran EMSAs to measure the affinities of the λ-CI protein for the six operator sites of λ-phage and the 12 binding sites of the other two lambdoid phages: VT2-SA and Stx2I. The resulting gels and analyses of the interactions of λ-CI to its six binding sites are shown in Figure 3 and Supplemental Figure S1, and the calculated *K_Dapp_* values are in Table 1. For the λ-phage operator sites, we observed that λ-O_R_1 (LOR1) and λ-O_L_1 (LOL1) have the highest apparent affinities, 0.35 ± 0.03 μM, and 0.21 ± 0.02 μM for the λ-CI, respectively, whereas λ-O_R_3 (LOR3) has the lowest affinity, 3.07 ± 1.13 μM [3,7,28,29,30,31]. 

**Figure 4:**
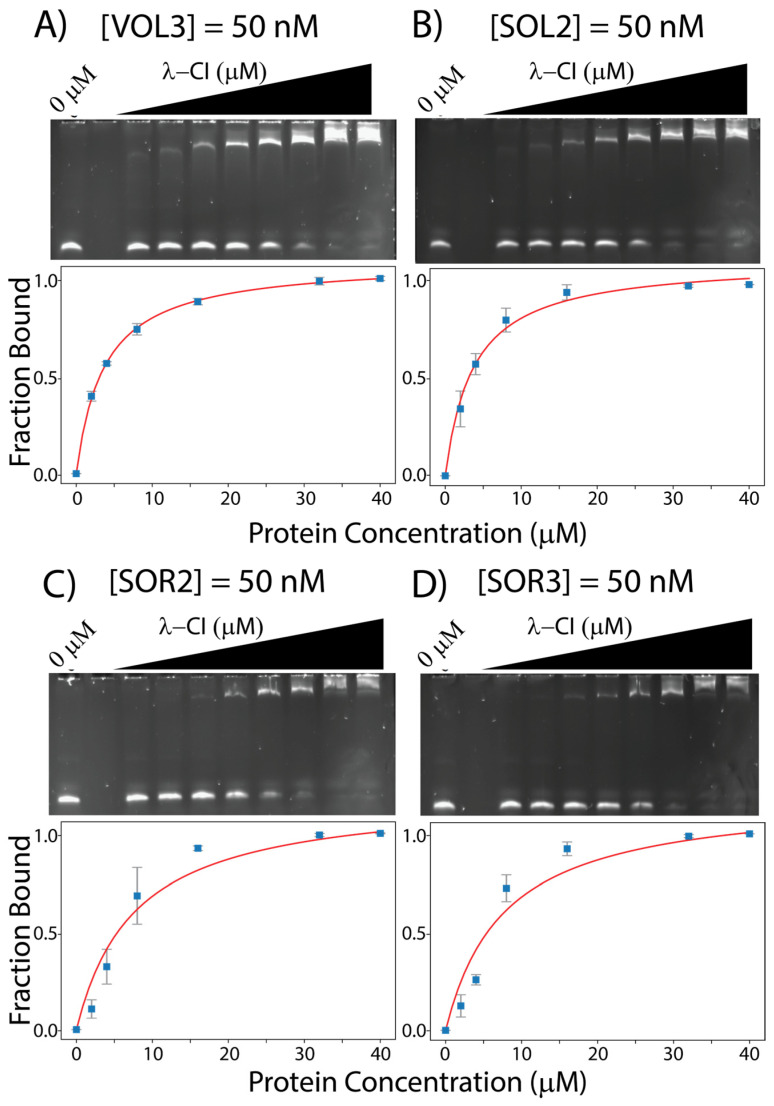
EMSAs gels for λ-CI with (**A**) 50 nM VOL3, (**B**) 50 nM SOL2, (**C**) 50 nM SOR2, and (**D**) 50 nM SOR3. All the binding data were measured using ImageJ and curve fitted using python.

For λ-CI binding to the other 12 operator sites from VT2-SA and Stx2-I, we observe VT2-SA-O_L_3 (VOL3) and Stx2I-O_L_2 (SOL2) have the highest apparent affinity for λ-CI (*K_Dapp_* values: 3.67 ± 0.10 μM and 3.68 ± 0.47 μM, Table 1), and Stx2I-O_R_3 (SOR3) and Stx2I-O_R_2 (SOR2) have the lowest apparent affinity for λ-CI (*K_Dapp_* values: 7.68 ± 3.10 μM and 7.67 ± 2.62 μM, Table 1). All the EMSAs between λ-CI and the operator sites for phage VT2-SA and Stx2-I are shown in Figure 4 and Supplemental Figures S2 and S3.

In the previous study, we extracted a distinct pattern of 14 bonds, and believe this represents the minimum direct readout information required by λ-CI to recognize its DNA-binding sites (Figure 2). Two hydrogen bonds within these 14 interactions, in locations 7D and 12A (Figure 2C), are maintained throughout all the sequences despite different nucleobases being present in those positions. We hypothesized that the interactions themselves are particularly important for binding. However, further analyses were needed to confirm this hypothesis.

In turn, we ran another in silico analysis for each of the 12 binding sites of phages VT2-SA and Stx2-I. We aligned each pattern of these 12 binding sites with the previously extracted pattern of λ-CI to calculate the percent-match (consensus %match) (Figures S4 and S5). After alignment, the resulting patterns were compared with the distinct pattern of λ-CI to see how many of the 14 bonds in the λ-CI distinct pattern were maintained in the six VT2-SA and six Stx2-I operator site patterns (Figures S6 and S7) and another distinct pattern match percentage—this one incorporating the published crystal structures—(distinct %match) was calculated for each of the 12 binding sites. 

**Figure 5:**
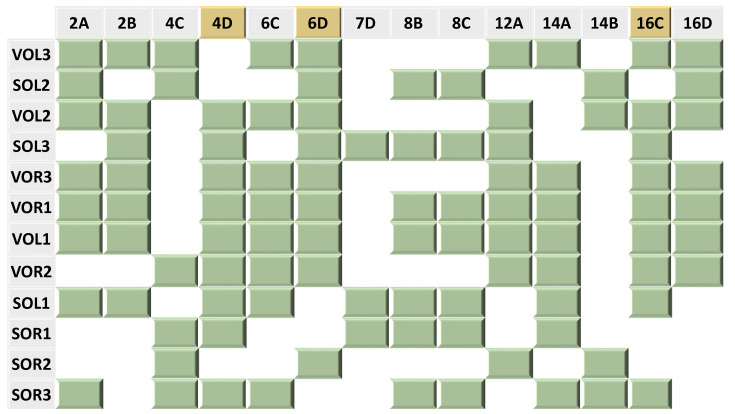
A map indicating the absence and presence of 14 distinct bonds in the 12 binding sites of the two phages: VT2-SA and Stx2I. The green blocks represent the presence of the corresponding bond in the corresponding binding sites while the white empty spaces represent the absence of the bonds. The bonds which are highlighted in yellow appeared in nine out of the 12 binding sites. Consult Figures S4–S7 for individual pattern alignments.

The 12 refined patterns are expressed in one map (Figure 5) which ranks the sites in descending order of affinity, based on their *K_Dapp_*, and examines which interactions are maintained. The refined pattern map indicates that the three bonds in positions 4D, 6D, and 16C are maintained in nine out of 12 binding sites, which refers to a possible role of these bonds in recognition. Additionally, we notice that position 12A is present in eight of the possible sites. This is in keeping with our previous hypothesis that this position is important for affinity. 

The in silico results indicate that the majority of the binding sites maintained at least 50% of either the consensus %match, distinct %match, or both (Table 2). However, the map accounting for individual interactions (Figure 5) shows that the binding sites which show a higher apparent affinity for λ-CI maintain at least one of the two distinct bonds: 6D and 12A (Figure 5). For example, VOL3 which has the highest affinity for λ-CI among the 12 non-λ operator sites, maintains the Purine N7 lone pair hydrogen bond acceptors at location 12A and location 6D (Figure 5, Table 2). On the other hand, SOR3, which has the lowest binding affinity for λ-CI, didn’t maintain either of the two distinct bonds (Figure 5, Table 2). It is noteworthy that the bond at position 7D is less important in recognition than expected, only showing up in three of the twelve sites; however, its presence contributes to the affinity of binding since SOL1 and SOR1, which both lack bonds 6D and 12A. These two sites have a higher apparent affinity for λ-CI compared to SOR3, which lacks all of the 7D, 6D, and 12A bonds (Figure 5, Table 2).

## 4. Discussion

Certain lambdoid phages can assume two lifecycles: a replicative lytic lifecycle that lyses the bacterial host or a relatively silent lysogenic lifecycle. Lysogen stability is primarily dependent on the CI repressor protein which binds six unique but related DNA sequences in the operator region. Our previous study focused on an algorithm that extracts the minimum information requirements of direct readout needed by λ-CI to recognize its multiple DNA binding sites. It is important to note that recognition of DNA from proteins is not solely dependent on the direct readout of the DNA major groove, and many other factors play a role in this process [32]. However, it has been shown that sequence-specificity is primarily derived from direct readout. Thus, we chose to focus on that in this study [17,33,34,35]. 

We note that our measured *K_Dapp_* values for the λ-CI for its natural binding partners do not match the established values, reported by Johnson et al., of 3 nM for O_R_1 and ~75 nM for O_R_2 and O_R_3 when measured independently [36]. This is partially due to the fact that we wanted a standard set of conditions across all of our samples that could capture the relative affinities and settled on 50 nM in our EMSA experiments, which is not capable of capturing the 3 nM binding constant. This discrepancy mainly arises from the fact that EMSA experiments do not necessarily measure thermodynamic equilibrium values due to dissociation and association that occur while the bands are running in the gel [37,38]. Additives can be mixed with the sample to minimize this, but we did not do this in our experiments as we did not want to disrupt the potentially weak binding of λ-CI to the operator DNA of the other two phages. We also did not minimize our gel-running times, gel dead-time, or vary the salt concentrations, which are other known ways of limiting protein–DNA dissociation within the gel itself. We feared that sample-to-sample variation would not allow for a direct comparison of affinities.

In a previous study, we used the operator sites of λ-phage towards our algorithm, and extracted the consensus pattern that is shared among these six binding sites. We further refined this pattern and removed extraneous contacts using the published crystal structure (1LMB) [39], resulting in an array of 14 bonds that represent the minimal information needed for recognition between λ-CI and its six binding sites. Two other members of the lambdoid phage family, VT2-SA and Stx2I, were used in that study. Their binding sites were aligned with those of the λ-phage to determine if any information might be shared amongst the three phages during evolution. These past results indicated a considerable amount of shared information among the three phages which led us to hypothesize that λ-CI could bind to the operator sites of these other phages: VT2-SA and Stx2I. In vitro data shows that all 12 binding sites bind the λ-CI (Table 1). 

Previously, we hypothesized that the two bonds—positions 7D and 12A—were important for λ-CI binding. We focused on these two hydrogen bond acceptors because they are maintained despite different nucleobase identities in the multiple operator sites. However, based on the data in Figure 5 and Table 2, we now hypothesize that the bonds at positions 6D and 12A are also important for binding. These bonds are present in nine of the 12 sequences. The interaction at position 7D is only present in three sequences, and in two of them, the hydrogen bond at 6D is missing. We hypothesize the hydrogen bond at 7D compensates if the interaction at position 6D is missing. For example, SOL1 and SOR1 both lack the 6D interaction but show a higher binding affinity for λ-CI compared to SOR3, which doesn’t maintain any of these interactions. Based on this analysis, and the in vitro data, we hypothesize that hydrogen bonds at positions 6D, 7D, and 12A are important for binding. SOR3 does not maintain any of these bonds and shows the lowest affinity among the 12 binding sites. This concept that single interactions can play a significant role in binding affinity has been seen in other unrelated systems [40]. 

Work from Fattah et al. previously pointed to the cross-immunity between VT2-SA and λ-phage, although the DNA sequence of the immunity region for both phages are dissimilar [11]. They also indicated, for the CI proteins of VT2-SA and λ-phage, a high amino acid sequence homology in the C-terminal dimerization region, and a low homology in the N-terminal DNA binding region. Lastly, they hypothesized that the few similarities in the recognition helices between λ-CI and VT2-SA-CI might be responsible for the cross-immunity shown in these phages [11]. 

Table 2 shows that, on an individual basis, λ-CI has the highest affinity for the VOR3 DNA-site. This would indicate that VT2-SA lysogeny would be weakened in co-infected *E. coli*, which Fattah et al. show is not the case. However, our data only looked at each operator site separately, which did not allow for cooperative binding which could explain this discrepancy. Coinfected strains would have both operator sites complete, and if the C-terminal sections of λ-CI and VT2-SA-CI are capable of cooperative binding, then the order of affinity in Table 2 would be altered. More work will have to be done to see if these two proteins cooperatively improve DNA binding as seen in λ-CI on its operator sites. 

By applying our algorithm to the operator sites of these phages, we found that λ-CI shows a relatively strong affinity to all the operator sites (i.e., almost 2-fold of LOR3-K_Dapp_) if they maintain at least one of the two bonds in positions 6D and 12A as well as greater than 50% of the distinct pattern (Table 2). Interestingly, these two positions of 6D and 12A appear to compensate for the loss of alternate bonds in distinct pattern. For example, SOR2, whose Distinct %match is 28.6%, has almost the same *K_Dapp_* value as SOR3, whose Distinct %match is 64.3%. The presence of these two bonds appears to compensate for the lack of bonds elsewhere in the major groove. However, we are unable to tell from this data if the sequence changes lead to other differences in protein or DNA structure that may bolster affinity. This finding emphasizes the significance of these two bonds in binding by λ-CI. By maintaining these two bonds and at least 50% of the distinct pattern from λ-phage’s six operator sites, λ-CI can bind DNA from other phages.

## 5. Conclusions

We believe that our algorithm is validated by the EMSA analyses presented throughout this manuscript and can predict the recognition and binding of certain DNA–protein combinations. However, the algorithm cannot estimate the *K_D_*, since direct readout is not the only factor that DNA-binding proteins depend on for recognition and specificity. Future work will delve into other factors in protein–DNA recognition, such as indirect readout and DNA methylation. 

## Figures and Tables

**Table 1:** Apparent dissociation constants (*K_Dapp_*) values for λ-CI binding individual 18 binding sites as measured using electrophoretic gel mobility assays. All values are reported in μM. All trials can be found in Tables S2–S19 in the Supplementary Data.

Operator Site	O_R_1	O_R_2	O_R_3	O_L_1	O_L_2	O_L_3
λ-Phage	0.35 ± 0.03	1.55 ± 0.29	3.07 ± 1.13	0.21 ± 0.02	1.50 ± 0.17	1.21 ± 0.08
VT2-SA	5.15 ± 2.39	6.39 ± 2.75	4.41 ± 0.99	5.37 ± 1.16	4.02 ± 0.16	3.67 ± 0.10
Stx2I	7.27 ± 2.39	7.67 ± 2.62	7.68 ± 3.09	7.11 ± 1.27	3.68 ± 0.47	4.26 ± 0.27

**Table 2:** Measurement of the apparent equilibrium dissociation constant (*K_Dapp_*) by gel mobility shift assay for λ-CI binding to the 12 binding sites of the other two phages, VT2-SA and Stx2I, their λ-consensus %match, λ-distinct %match, and the presence of the specific bonds at locations 6D, 7D, and 12A in their overlapped binding patterns.

	*K_Dapp_* (µM)	Consensus %Match	Distinct %Match	Specific Bonds
VOL3	3.67 ± 0.10	73.9%	64.3%	6D, 12A
SOL2	3.68 ± 0.47	43.5%	50.0%	6D
VOL2	4.02 ± 0.16	78.3%	64.3%	6D, 12A
SOL3	4.26 ± 0.27	52.2%	57.1%	6D, 7D, 12A
VOR3	4.41 ± 0.99	69.6%	64.3%	6D, 12A
VOR1	5.15 ± 2.39	73.9%	78.6%	6D, 12A
VOL1	5.37 ± 1.16	73.9%	78.6%	6D, 12A
VOR2	6.39 ± 2.75	65.2%	57.1%	6D, 12A
SOL1	7.11 ± 1.27	69.6%	64.3%	7D
SOR1	7.27 ± 2.39	39.1%	42.9%	7D
SOR2	7.67 ± 2.62	26.1%	28.6%	6D, 12A
SOR3	7.68 ± 3.09	65.2%	64.3%	None

## Data Availability

The genomic sequences of λ-phage, VT2-SA phage, and Stx2I phage’s binding sites are available online in the NCBI taxonomy database [25,26]. The code for our algorithm can be found in Sedhom, Kinser, Solomon 2022 [4].

## Supplementary Materials

The following supporting information can be downloaded at https://www.mdpi.com/article/10.3390/genes14122221/s1: Figure S1: EMSA gels for lambda CI and lambda operator sites; Figure S2: EMSA gels for lambda CI and VT2-SA operator sites; Figure S3: EMSA gels for lambda CI and Stx-2I operator sites. Table S1: sequences used of all DNA oligos discussed in this manuscript; Tables S2–S19: analysis data for EMSA gels noting the fraction of DNA bound per lane in each trial.
