# Supplementary material for: Lambda CI Binding to Related Phage Operator Sequences Validates Alignment Algorithm and Highlights the Importance of Overlooked Bonds
Source: Genes (Basel). 2023 Dec 15;14(12):2221. doi: 10.3390/genes14122221 (PMC10742460; doi:10.3390/genes14122221)
Supplement: Supplementary file 1 [file genes-14-02221-s001.zip › genes-2714088-supplementary.pdf]

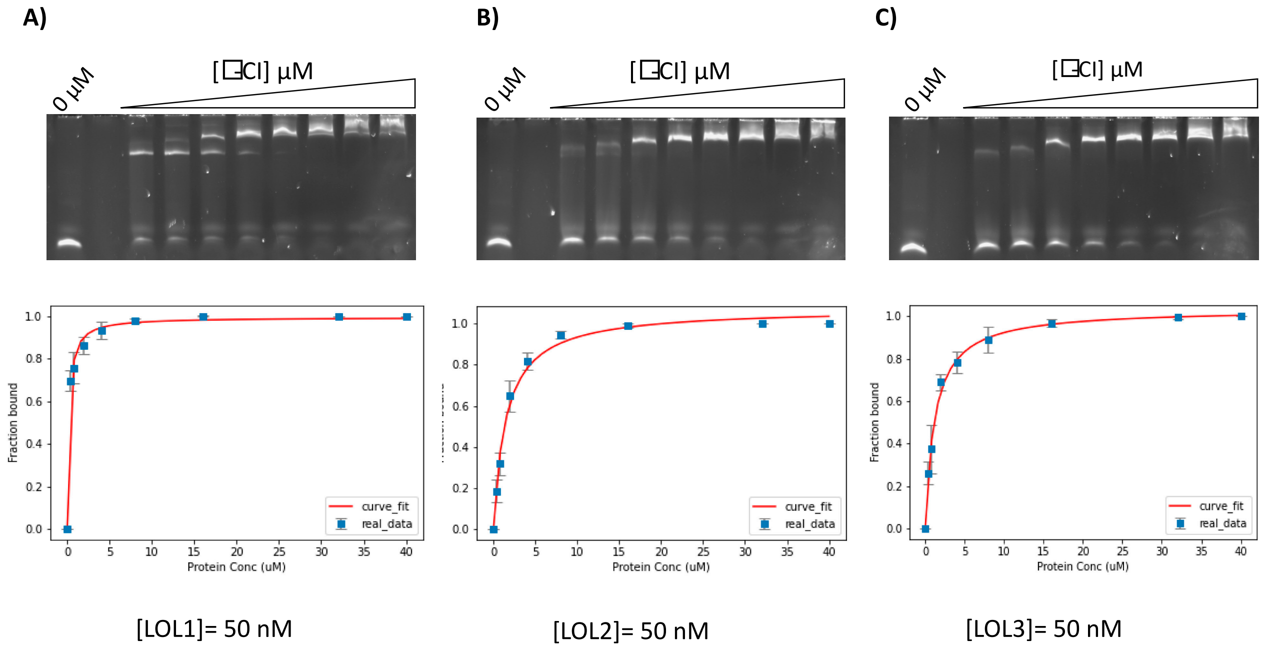

Figure S1. EMSAs gels for λ-CI with A) 50 nM LOL1, B) 50 nM LOL2 and C) 50 nM LOL3. All the binding data were measured using ImageJ and curve fitted using python.

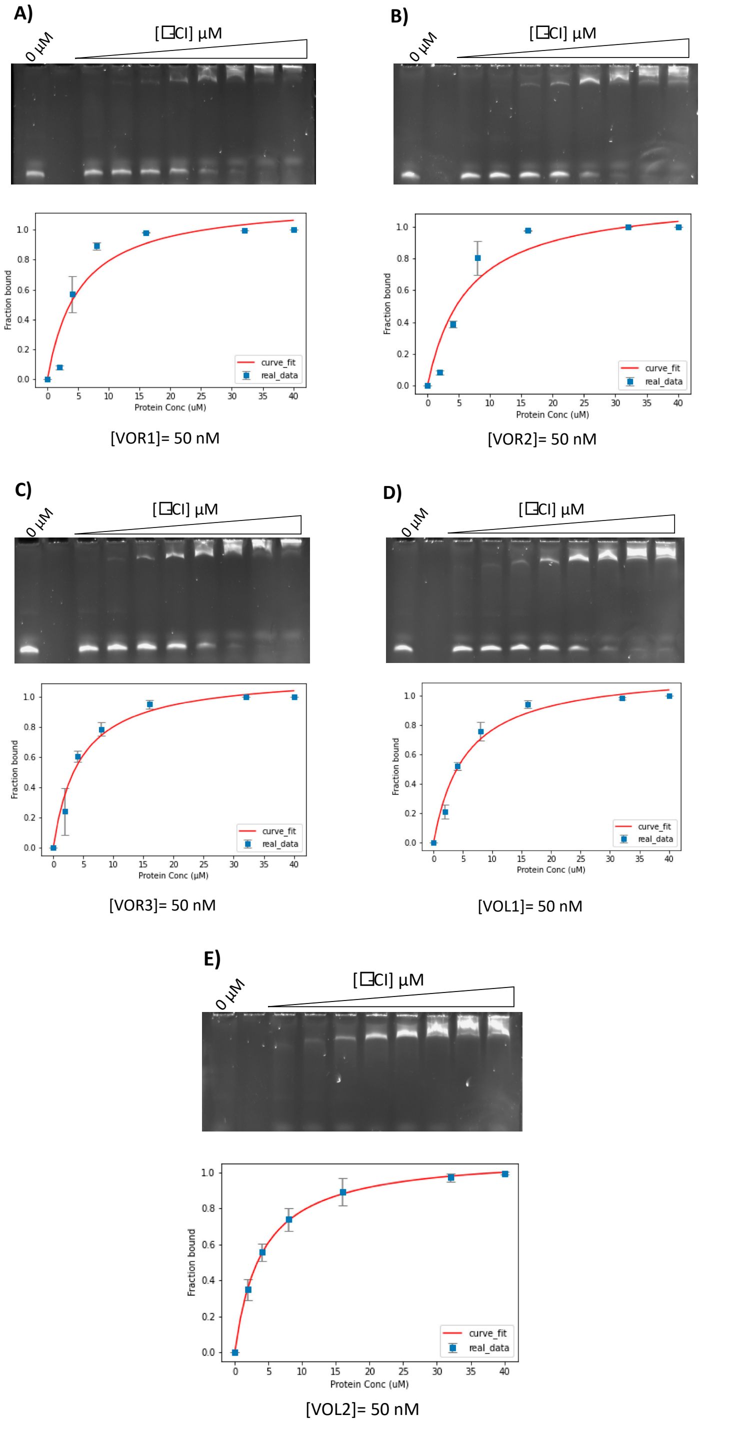

Figure S2. EMSAs gels for λ-CI with A) 50 nM VOR1, B) 50 nM VOR2, C) 50 nM VOR3, D) 50 nM VOL1 and E) 50 nM VOL2. All the binding data were measured using ImageJ and curve fitted using python.

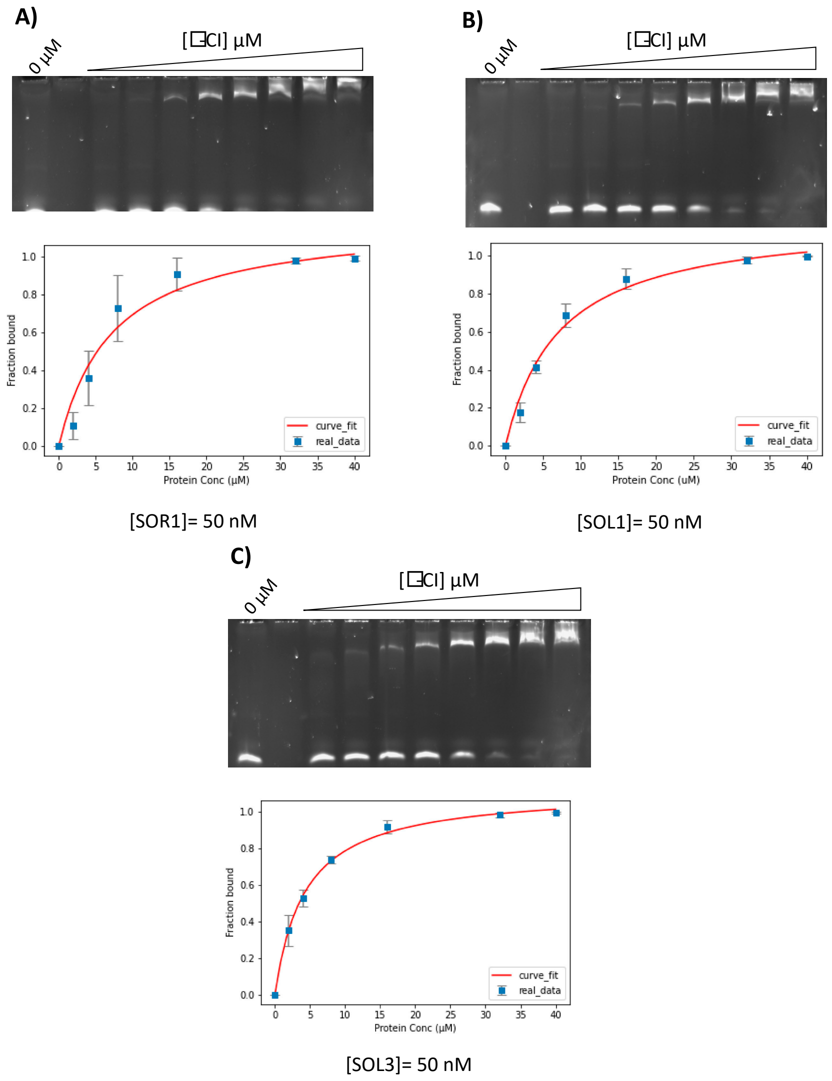

Figure S3. EMSAs gels for λ-CI with A) 50 nM SOR1, B) 50 nM SOL1 and C) 50 nM SOL2. All the binding data were measured using ImageJ and curve fitted using python.

Table S1. The DNA sequences of the three phages' binding sites in black. Overhang sequences are in green.

Phage Strain	Binding site	DNA Sequence
Lambda (λ)	OR1	GGTTATTATGG TACCTCTGGCGGTGATA TGCAAGTGC
	OR2	GGTTATTATGG TAACACCGTGCGTGTTG TGCAAGTGC
	OR3	GGTTATTATGG TATCACCGCAAGGGATA TGCAAGTGC
	OL1	GGTTATTATGG TATCACCGCCAGTGGTA TGCAAGTGC
	OL2	GGTTATTATGG CAACACCGCCAGAGATA TGCAAGTGC
	OL3	GGTTATTATGG TATCACCGCAGATGGTT TGCAAGTGC
VT2-SA	OR1	GGTTATTATGG AAGTACATATTGTATTT TGCAAGTGC
	OR2	GGTTATTATGG ATGCACAATGTGTATTT TGCAAGTGC
	OR3	GGTTATTATGG TATTACGCTCTGTACTG TGCAAGTGC
	OL1	GGTTATTATGG TAGTACGATATGTACTT TGCAAGTGC
	OL2	GGTTATTATGG CAATACAATTTGTTCTA TGCAAGTGC
	OL3	GGTTATTATGG TACAACGCTTTGTATTT TGCAAGTGC
Stx2I	OR1	GGTTATTATGG TTTCAGTTCAACCATAA TGCAAGTGC
	OR2	GGTTATTATGG ATGAGTACGATACTAAA TGCAAGTGC
	OR3	GGTTATTATGG GGGCGAATTCTTTGCGC TGCAAGTGC
	OL1	GGTTATTATGG CAATGATGATTTCATGC TGCAAGTGC
	OL2	GGTTATTATGG TGTAATATTTTCTTACA TGCAAGTGC
	OL3	GGTTATTATGG GCTTCTTGAATAACAGC TGCAAGTGC

Table S2. The binding data for λ-CI with LOR1						
CI conc (uM)	trials				average	SD
	1	2	3	4		
0	0	0	0	0	0	0
0.4	0.64215724	0.63714968	0.36051448		0.54660713	0.16118041
0.8	0.81082533	0.80156855	0.62118743	0.51932969	0.68822775	0.14247487
2	0.95705854	0.93095137	0.90621801	0.86012149	0.91358735	0.04124786
4	0.97754402	0.9889962	0.96971053	0.96144871	0.97442487	0.01172829
8	0.98305379	0.99586596	0.99014985	0.98752654	0.98914903	0.00535111
16	0.99659658	1	0.99738435	0.99077755	0.99618962	0.00389027
32	1	1	1	1	1	0
40	1	1	1	1	1	0

Table S3. The binding data for λ-CI with LOR2								
CI conc (uM)	trials						average	SD
	1	2	3	4	5	6		
0	0	0	0	0	0	0	0	0
0.4	0.10279775	0.08526245	0.14515904	0.12326493	0.13748501	0.11015927	0.11735474	0.02239122
0.8	0.32027149	0.41100334	0.20627587	0.19479572	0.47697192	0.19163987	0.3001597	0.12300053
2	0.73205124	0.73126619	0.56563037	0.71177839	0.65448723	0.67827907	0.67891541	0.06336345
4	0.90328677	0.93873148	0.88492019	0.78289039	0.6949698	0.88846965	0.84887805	0.09158886
8	0.9662105	0.99088871	0.94249541	0.9235707	0.98185285	0.97730478	0.96372049	0.02576873
16	0.99545487	0.99892665	0.95906681	0.97708132	0.99000016	0.99346508	0.98566582	0.01504986
32	1	1	1	1	1	1	1	0
40	1	1	1	1	1	1	1	0

Table S4. The binding data for λ-CI with LOR3						
CI conc (uM)	trials				average	SD
	1	2	3	4		
0	0	0	0	0	0	0
0.4	0.01074645	0.02371539	0.0429321	0.03378716	0.02779528	0.01381224
0.8	0.08518358	0.06505735	0.083499	0.04957068	0.07082765	0.01685043
2	0.37680368	0.30580252	0.31940157	0.35947863	0.3403716	0.03330234
4	0.86521344	0.81709052	0.84190748	0.86737039	0.84789546	0.02355126
8	0.99583305	0.96966002	0.97585521	0.97558424	0.97923313	0.01142988
16	1	1	1	1	1	0
32	1	1	1	1	1	0
40	1	1	1	1	1	0

Table S5. The binding data for λ-CI with LOL1						
CI conc (uM)	trials				average	SD
	1	2	3	4		
0	0	0	0	0	0	0
0.4	0.74275585	0.73421081	0.66133934	0.64591473	0.69605518	0.04951787
0.8	0.81615543	0.8250863	0.68089326	0.70580733	0.75698558	0.07426985
2	0.8781074	0.91176577	0.83248591	0.82143733	0.8609491	0.04182533
4	0.97661281	0.93991034	0.88264283	0.92777798	0.93173599	0.03875802
8	0.98323567	0.98496397	0.96620679	0.9741487	0.97713878	0.00869587
16	1	1	1	0.99544462	0.99886115	0.00227769
32	1	1	1	1	1	0
40	1	1	1	1	1	0

Table S6. The binding data for λ-CI with LOL2						
CI conc (uM)	trials				average	SD
	1	2	3	4		
0	0	0	0	0	0	0
0.4	0.23799133	0.22091393	0.16967566	0.11503125	0.18590304	0.05545295
0.8	0.33608317	0.33083302	0.3667847	0.24084449	0.31863635	0.05423095
2	0.57557648	0.69539164	0.58713738	0.7317327	0.64745955	0.07790034
4	0.76388376	0.8240741	0.86713628	0.81002573	0.81627997	0.04255033
8	0.9615783	0.92528135	0.93196017	0.96090744	0.94493182	0.01903269
16	0.99231078	0.9777523	0.98439195	1	0.98861376	0.00964554
32	1	1	1	1	1	0
40	1	1	1	1	1	0

Table S7. The binding data for λ-CI with LOL3						
CI conc (uM)	trials				average	SD
	1	2	3	4		
0	0	0	0	0	0	0
0.4	0.30476998	0.30763117	0.19783118	0.23669997	0.26173307	0.05375537
0.8	0.41190544	0.43094454	0.20352838	0.45163638	0.37450368	0.11513249
2	0.64275015	0.72500806	0.67464069	0.71664094	0.68975996	0.03831244
4	0.76551701	0.74636287	0.7682345	0.86030706	0.78510536	0.05107056
8	0.8120679	0.87721704	0.94002501	0.93063476	0.88998618	0.05885151
16	0.94658288	0.96583899	0.98879278	0.97235494	0.9683924	0.0174552
32	1	0.98430365	1	1	0.99607591	0.00784817
40	1	1	1	1	1	0

Table S8. The binding data for λ-CI with VOR1						
CI conc (uM)	trials				average	SD
	1	2	3	4		
0	0	0	0	0	0	0
0.4	0.01402669	0.01181229	0.00889833	0.01088209	0.01140485	0.00212885
0.8	0.02997044	0.03306916	0.03822149	0.02695978	0.03205522	0.00480835
2	0.07618689	0.09965214	0.06991182	0.07678799	0.08063471	0.01305403
4	0.53991689	0.4101596	0.67461383	0.64890473	0.56839876	0.12057322
8	0.92098892	0.86184614	0.89215216	0.89052237	0.8913774	0.02415415
16	0.97992512	0.98719576	0.98117429	0.97780621	0.98152534	0.0040278
32	0.99667168	0.99487729	0.99319429	0.996975	0.99542956	0.0017543
40	1	1	1	1	1	0

Table S9. The binding data for λ-CI with VOR2						
CI conc (uM)	trials				average	SD
	1	2	3	4		
0	0	0	0	0	0	0
0.4	0	0	0	0	0	0
0.8	0.02057431	0.02229398	0.01539598	0.00478789	0.01576304	0.00788226
2	0.08824867	0.06646483	0.09858852	0.0820022	0.08382606	0.01344406
4	0.41114593	0.36050232	0.39504381	0.37904633	0.3864346	0.02169361
8	0.64663339	0.87280696	0.85236506	0.85185881	0.80591605	0.10663584
16	0.97295743	0.96887687	0.98230809	0.97825865	0.97560026	0.00589493
32		0.99704761	0.99495211	0.97481748	0.98893907	0.01227445
40		1	1	0.98318958	0.99439653	0.0097055

Table S10. The binding data for λ-CI with VOR3						
CI conc (uM)	trials				average	SD
	1	2	3	4		
0	0	0	0	0	0	0
0.4	0	0	0	0	0	0
0.8	0.04886266	0.02413568	0.04429557	0.04827195	0.04139147	0.01168126
2	0.11815051	0.11920422	0.44086945	0.28888996	0.24177853	0.15509678
4	0.55060737	0.6220591	0.62070109	0.63122297	0.60614763	0.03732055
8	0.74041737	0.77285962	0.84720425	0.7840791	0.78614008	0.04472143
16	0.96392937	0.91393197	0.97830775	0.96667963	0.95571218	0.02854209
32	0.95861666	0.95324985	0.99652787	0.99295673	0.97533778	0.02256043
40		1	1	1	1	0

Table S11. The binding data for λ-CI with VOL1						
CI conc (uM)	trials				average	SD
	1	2	3	4		
0	0	0	0	0	0	0
0.4	0.03057005	0.05819495	0.02850897	0.02397533	0.03531232	0.01550179
0.8	0.07160596	0.10721945	0.09607109	0.07593393	0.08770761	0.01681807
2	0.17766816	0.26756022	0.22409557	0.17032692	0.20991272	0.04520743
4	0.48212511	0.51637814	0.53282156	0.54807347	0.51984957	0.02828455
8	0.70521508	0.8244549	0.70404005	0.80055965	0.75856742	0.06304552
16	0.94163359	0.97338456	0.8784265	0.93045004	0.93097367	0.03947065
32	0.98056496	0.98508735	0.97074512	0.98298215	0.9798449	0.00634166
40		1	0.98956712	0.98955347	0.9930402	0.00602737

Table S12. The binding data for λ-CI with VOL2						
CI conc (uM)	trials				average	SD
	1	2	3	4		
0	0	0	0	0	0	0
0.4	0.08000414	0.08056843	0.11537065	0.06226504	0.08455207	0.02223398
0.8	0.14845294	0.11181472	0.14178042	0.09284196	0.12372251	0.02603258
2	0.40916511	0.26656848	0.36058305	0.35906424	0.34884522	0.05958231
4	0.52267241	0.51340244	0.61217308	0.58201066	0.55756465	0.04742523
8	0.66298645	0.71520899	0.81242318	0.76559397	0.73905315	0.06440047
16	0.83293489	0.82922647	0.93710878	0.97912333	0.89459837	0.07533807
32	0.94858903	0.95762087	0.9848738	1	0.97277093	0.02382009
40		0.98826482		1	0.99413241	0.00829802

Table S13. The binding data for λ-CI with VOL3						
CI conc (uM)	trials				average	SD
	1	2	3	4		
0	0	0	0	0	0	0
0.4	0.11351357	0.11800529	0.09511538	0.03921621	0.09146261	0.03621136
0.8	0.1928493	0.22432755	0.22305369	0.17858345	0.2047035	0.02269076
2	0.40941596	0.41946489	0.37259066	0.3713141	0.3931964	0.02487662
4	0.5644268	0.55823846	0.56719082	0.54784369	0.55942494	0.00858022
8	0.6946271	0.75325841	0.72598929	0.75732082	0.73279891	0.02900214
16	0.88021251	0.88280774	0.84857208	0.88110808	0.8731751	0.01643729
32	1	0.9538181	0.97302409	0.98232416	0.97729159	0.01923725
40	1	0.97942233	1	0.98440898	0.99095783	0.01063762

Table S14. The binding data for λ-CI with SOR1						
CI conc (uM)	trials				average	SD
	1	2	3	4		
0	0	0	0	0	0	0
0.4	0	0	0	0.01947984	0.00486996	0.00973992
0.8	0.01304342	0.04837218	0.0538849	0.0574276	0.04318203	0.02043495
2	0.0402713	0.08397515	0.20851674	0.10078474	0.10838698	0.07145965
4	0.3027054	0.19663327	0.52325953	0.41840329	0.36025038	0.14146345
8	0.5000063	0.68899767	0.89545658	0.82701548	0.72786901	0.17449739
16	0.82689133	0.83896227	0.98386784	0.98746369	0.90929628	0.08833367
32	0.9617668	0.97551262	0.99444645	0.99496582	0.98167292	0.01606307
40	0.9779765	1	1	1	0.99449412	0.01101175

Table S15. The binding data for λ-CI with SOR2						
CI conc (uM)	trials				average	SD
	1	2	3	4		
0	0	0	0	0	0	0
0.4	0	0	0	0	0	0
0.8	0.00592222	0.02254033	0	0	0.00711564	0.01065536
2	0.07986793	0.11951658	0.05727472	0.16368442	0.10508591	0.04677565
4	0.26374299	0.38601725	0.40590062	0.22666082	0.32058042	0.08871847
8	0.46515866	0.75354023	0.76322201	0.74266345	0.68114609	0.14423629
16	0.90565095	0.92694548	0.93200518	0.92711663	0.92292956	0.01175552
32	0.98106216	0.99225687	1	0.98680554	0.99003114	0.00806596
40	1	1	1	0.9950606	0.99876515	0.0024697

Table S16. The binding data for λ-CI with SOR3

CI conc (uM)	trials				average	SD
	1	2	3	4		
0	0	0	0	0	0	0
0.4	0	0	0	0	0	0
0.8	0.0263119	0.0322789	0.02467223	0.02234692	0.02640249	0.00424193
2	0.07515644	0.10437324	0.11292587	0.20621888	0.12466861	0.05672065
4	0.25803062	0.22253041	0.28552586	0.26466536	0.25768806	0.02620308
8	0.71389634	0.6372837	0.74045782	0.80179675	0.72335865	0.06817466
16	0.95229108	0.94763626	0.87673028	0.91823087	0.92372212	0.03476808
32	1	0.98218216	0.98313518	0.98713873	0.98811402	0.00820982
40	1	1	1	1	1	0

Table S17. The binding data for λ-CI with SOL1

CI conc (uM)	trials				average	SD
	1	2	3	4		
0	0	0	0	0	0	0
0.4	0.01463321	0	0	0	0.0036583	0.0073166
0.8	0.05317782	0.03911125	0.02728853	0.02268064	0.03556456	0.01362955
2	0.1501219	0.20956129	0.22729639	0.11753331	0.17612823	0.05113917
4	0.36805867	0.44948844	0.42042621	0.41620982	0.41354578	0.03374112
8	0.66252366	0.61674738	0.76790415	0.69628315	0.68586459	0.06366847
16	0.86342997	0.81569019	0.88523374	0.94945947	0.87845334	0.05553605
32	1	0.95291522	0.97682745	0.97735973	0.9767756	0.01922702
40	1	1	0.99311855	0.99574557	0.99721603	0.00338883

Table S18. The binding data for λ-CI with SOL2						
CI conc (uM)	trials				average	SD
	1	2	3	4		
0	0	0	0	0	0	0
0.4	0.0906104	0.03082151	0.05562639	0.02286552	0.04998096	0.03046831
0.8	0.0891321	0.0933386	0.08992544	0.06878079	0.08529423	0.0111592
2	0.39913809	0.22492358	0.4390636	0.33564389	0.34969229	0.09344644
4	0.61427868	0.50909633	0.63514091	0.57753222	0.58401204	0.05533107
8	0.86265941	0.72272238	0.83910715	0.83233576	0.81420618	0.06235848
16	0.90159999	0.96373558	0.99163682	0.97925797	0.95905759	0.03996966
32	0.98467916	0.99040399	1	0.99443241	0.99237889	0.00646747
40	1	1	1	1	1	0

Table S19. The binding data for λ-CI with SOL3						
CI conc (uM)	trials				average	SD
	1	2	3	4		
0	0	0	0	0	0	0
0.4	0.12759012	0.09792431	0.0715258	0.03365832	0.08267464	0.03990342
0.8	0.20679682	0.13688742	0.11548178		0.15305534	0.04775626
2	0.43725049	0.41679413	0.2863562	0.27681588	0.35430418	0.08447179
4	0.54367471	0.58354003	0.47767705	0.50614501	0.5277592	0.04597173
8	0.74873527	0.7451234	0.75414504	0.70663891	0.73866065	0.02166736
16	0.87716312	0.95709068	0.92910767	0.91189007	0.91881289	0.03343551
32	1	0.98414138	0.98647421	0.96934697	0.98499064	0.01255558
40	1	0.98994859	0.9926234	1	0.995643	0.00514818
